# How do physicians and nurses in family practice describe their care for patients with progressive life-limiting illness? A qualitative study of a ‘palliative approach’

**DOI:** 10.1017/S1463423619000252

**Published:** 2019-07-01

**Authors:** Alex Rewegan, Sharef Danho, Joy White, Samantha Winemaker, Nicolle Hansen, Amanda MacLennan, Michelle Howard

**Affiliations:** 1 Department of Social Anthropology, York University, Toronto, Ontario, Canada; 2 Department of Family Medicine, McMaster University, Hamilton, Ontario, Canada; 3 Faculty of Medicine, University of Toronto, Toronto, Ontario, Canada

**Keywords:** primary care, family practice, palliative care medicine, qualitative research, chronic illness, end of life

## Abstract

**Aim:**

To explore how a palliative approach to care is operationalized in primary care, through the description of clinical practices used by primary care clinicians to identify and care for patients with progressive life-limiting illness (PLLI).

**Background:**

Increasing numbers of people are living with PLLI but are often not recognized as needing a palliative approach to care. To meet growing needs, generalists such as family physicians will need to adopt a palliative approach to care in their own setting. Practical descriptions of a palliative approach in non-specialist settings have been lacking.

**Methods:**

We conducted a qualitative descriptive study design using in-depth semi-structured interviews with 11 key informant participants (6 physicians, 3 nurse practitioners, 1 registered nurse, and 1 registered practical nurse) known to be providing comprehensive care to patients with PLLI in family practices in Ontario, Canada. We asked about their approach to identifying patients with PLLI and the strategies used in their care. We employed content analysis to develop themes.

**Findings:**

Participants identified patients by functional decline, change in needs, increased acuity, and the specifics of a condition/diagnosis. Care strategies included concretizing commitment to care, eliciting goals of care, shifting care to the home, broadening team members including leveraging the support of family and community resources, and shifting to a ‘proactive’ approach involving increased follow-up, flexibility, and intensity.

**Conclusion:**

Primary care providers articulated strategies for identifying and providing care to patients with PLLI that illuminate an upstream approach tailored to their setting.

## Introduction

Palliative care is defined as the active holistic care of individuals across all ages with serious health-related suffering due to severe illness and especially of those near the end of life. It aims to improve the quality of life of patients, their families, and their caregivers (IAHPC, 2018). Our aging demographics and increasing prevalence of chronic diseases will intensify the needs of health care systems to provide such palliative forms of care. Worldwide, approximately 40 million people annually are in need of palliative care, and it has been estimated that as few as 14% of people receive formal palliative care (World Health Organization, 2017). In Canada, one-quarter of a million die each year, and by 2036, this number is expected to double (Fowler *et al*., 2013). Importantly, most of these people will die of progressive life-limiting illnesses (PLLIs), and few will die suddenly (Lunney *et al*., 2003; Fassbender *et al*., 2009; Aldridge & Bradley, 2017).

Multiple randomized trials and comparative studies have shown that compared with usual care, palliative care is associated with better patient and system outcomes including improved quality of life, symptom burden, family distress, satisfaction with care, decreases in use of higher acuity health services and costs (Smith *et al*., 2015), and has even improved survival (Temel *et al*., 2010). Palliative care, however, is more often provided to individuals with cancer than other end-of-life trajectories such as organ failure, frailty, and dementia (Seow *et al*., 2014; Seow *et al*., 2018), and a large proportion of people – especially those with non-cancer diagnoses – never receive palliative care (Tanuseputro *et al*., 2017; Seow *et al*., 2018). There is in turn an increasing recognition of the need for palliative care among patients with non-cancer illnesses (Lunney *et al*., 2003; Stajduhar, 2011; Gómez-Batiste *et al*., 2017), where medically definable boundaries between living and dying are often ambiguous and in tension (Bern-Klug, 2009; Banerjee & Rewegan, 2017).

In response to this changing epidemiological and demographic context – with people living longer and with more complex, compounded illnesses – dominant conceptualizations of palliative care as a specialty discipline, with care only provided at the very end of life, have needed to adapt and expand to the clinical practice domain of generalists, woven through ongoing primary care (Shadd *et al*., 2013; Canadian Hospice Palliative Care Association, 2014; National Institute for Health and Care Excellence, 2015; Sawatzky *et al*., 2016; Gómez-Batiste *et al*., 2017). The central role of family physicians and primary care settings in providing such palliative care has been widely recognized and advocated. However, the adaptation of elements of palliative care to leverage primary care and other generalist clinicians has not been well operationalized (Shadd *et al*., 2013; Reyniers *et al*., 2014; Beernaert, Deliens, *et al*., 2015). Efforts to integrate palliative care in and across health care systems, as recommended by the World Health Organization (Canadian Hospice Palliative Care Association, 2013; Sawatsky *et al*., 2016), have been slow to achieve uptake and sustainability (Shaw *et al*., 2010; Tapsfield *et al*., 2016; Murray *et al*., 2017).

In response to the challenges of integrating palliative care that have arisen due to specialization, attempts have been made to gain more conceptual clarity of the elements of a ‘palliative approach’ that could be infused throughout health care settings. A systematic review described the conceptual elements of a primary ‘palliative approach’ as (1) an upstream orientation toward the needs of people and their families who live with life-limiting illness, early on and throughout the illness trajectory, (2) the adaptation and iteration of palliative care knowledge and expertise to varying illnesses and situations, (3) and the operationalization of such a palliative approach through its integration into systems and models of care that do not specialize in palliative care (Sawatzky *et al*., 2016). Further, in an attempt to bridge the philosophy of a palliative approach with specific clinical care, another review summarized the common domains of definitions of palliative care or a palliative approach, as care that simultaneously addresses whole-person needs, enhances quality of life, and acknowledges mortality (Touzel and Shadd, 2018). Together these works provide a conceptualization of a palliative approach that can guide any health care setting.

Our aim in this study was to provide a description, from the perspective of ‘key informants’, of how elements of a palliative approach were operationalized through clinical behaviors, performances, and practices by non-specialist primary care clinicians. To help move existing conceptual descriptions toward an understanding of such everyday clinical processes, we conducted a qualitative descriptive study of how clinicians in primary care enact, negotiate, and adapt a palliative approach for their patients with PLLI.

## Methods

### Study design

We chose a qualitative descriptive study design given the exploratory nature of the research, as well as the need to provide an in-depth descriptive account of the phenomenon (Neergaard *et al*., 2009). Central to our inquiry was the idea that questioning clinicians about their provision of ‘palliative care’ would narrow the responses to be about care of dying patients at the very end of life. Consistent with current conceptions of a palliative approach for people with life-limiting illness that are not tied to a specific diagnosis, trajectory, or expected proximity to end of life, we framed our questions around care for patients with ‘PLLI’. We did not explicitly ask participants how they provide palliative care because the term is generally used to refer to the field of specialist palliative medicine.

### Sample selection

From January to April 2015, two clinician members of the research team (S.W., A.M.) initially recruited primary care clinicians (physicians and nurses) in Hamilton and the Greater Toronto Area, Ontario, whom they identified as representing key informants in their medical communities. Key informants, due to their unique authorial and/or skilled position within a group or society, are able to observe and articulate deeper insights into the nuances of their surroundings and the internal working dynamics of their peer groups. Marshall describes these individuals as ‘natural observers’, people who are inquisitive about the culture and behavior of their communities, and who are particularly observant of continuity and change (Marshall, 1996). By using a key informant recruitment strategy, we sought out clinicians who members of our research team and their associated medical communities already recognized as provisioning care in ways that are consistent with existing conceptualizations of a primary palliative approach for their patients with PLLI.

Clinician team members N.H. and J.W. – initially recruited as key informants – subsequently joined our research team as both participants and researchers (see below). All clinician members of the research team (N.H., J.W., A.M., and S.W.) then worked to further identify nine additional clinicians active in their daily working environments who they considered to be providing forms of care congruent with emerging conceptions of a primary palliative approach. Including our two key informant research team members, we ultimately recruited a total of 11 key informants. We sought variation in profession among participants (physician, nurse, nurse practitioner) and practice environment (group versus solo physician), and all participants worked in different practices from one another. All candidates responded to an invitation, provided written informed consent, and participated in a semi-structured interview.

Our decision to include our first two key informants as members of the research team is consistent with forms of qualitative research like participatory action research and ethnographic methodologies that seek to leverage the situated knowledge and local perspectives of research subjects in the development and reflexive iteration of research design and analysis (Cornwall & Jewkes, 1995). By doing so, we wanted to ensure that our key informant methodology would elicit interviews that aligned sharply with forms of care relevant to a primary palliative approach while keeping our interview guide, coding, and thematic analyses informed by the experiential knowledge of our team members, primary care clinicians who are grappling with the need to provide palliative forms of care to a growing population of patients with PLLI in their practices.

### Data collection

The interviewer (A.R.) asked the participants a series of semi-structured questions following an interview guide. Through an iterative process informed by current conceptualizations of a palliative approach, we compiled the interview guide by brainstorming and coming to consensus on a set of open-ended questions (Table 1). We specifically wanted to gain insight into how clinicians identify patients with PLLI and the strategies used to provide their care. Additionally, during the interview, the interviewer (A.R.) worked to elicit discussion and probe responses about the positive and promising care practices that our participants believed led to patients, family, and themselves feeling successful and fulfilled by their care.

**Table 1.** Semi-structured interview guide

Please imagine a patient from your practice with complex health needs. Ensure that this patient represents someone who is/was experiencing PLLI from which they will not recover. Keep this patient in mind – and your experiences with them – to help inform your answers to the questions asked throughout the interview. Feel free to think of numerous patient cases. Broadly, we will be interested in your approaches to meeting the needs of these patients throughout their illness trajectories.
**Definition and Identification**
How might you and your practice define a “progressive life-limiting illness”? How do you and your practice identify patients with this form of illness?
**Recognition and Planning**
How do you and your practice recognize, and plan for, patient specific needs? What type of patients are they, and what triggers your recognition of a progressive life-limiting illness? What strategies and practices do you use to discuss these needs with patients?
**Care Strategies**
What strategies do you and your practice use to support these patients? How do you and your practice define support? What about the patients’ family/caregiver?
**Practical and Examples**
What about your practice would demonstrate that care and support of patients with progressive life-limiting illness is taking place? Does your practice organize itself around the care of these patients, and in what ways?

We tested the initial interview guide by conducting our first two interviews with our key informant research team members (N.H. and J.W.) in order to gauge its length, feasibility, and relevance. We then iterated the guide through minor changes in language and probing questions for use in the subsequent nine key informant interviews.

All interviews took place in participants’ active practice/workplace. An audio recording device recorded the interviews, averaging 40 minutes in length. The interviewer transcribed the recordings verbatim. The Hamilton Integrated Research Ethics Board approved this study.

### Data analysis

We employed content analysis to develop a thematic framework that would allow for rich description of our participants’ care practices for patients with PLLI (Hsieh & Shannon, 2005; Charmaz, 2014). Two interviews were first reviewed by two research team members who created the preliminary coding framework (A.R. and S.D.). Among the initial transcripts, we asked a student research assistant to select a transcript from a participant working in a team-based family academic practice and one working in a solo community practice. All team members then worked individually to code a third interview using this preliminary coding framework. The full team then finalized the framework with new and previously unidentified themes. We applied the revised framework by coding all 11 interviews using NVivo 10 software (QSR International, 2012). The team then met every two weeks for 3 months to refine codes and to identify and articulate recurring themes and sub-themes by working through each transcript line by line. We then grouped recurring themes into the broader categories used for interpretation and synthesis of the results.

After coding nine interviews, no new main themes emerged, though we continued to code the remaining two interviews to ensure sufficient saturation. While saturation is often an important feature of qualitative research, our goal was not to achieve an exhaustive or totalizing account of primary care practice but to highlight some of the promising practices used by key informants that warrant future research and exploration in conceptualizing a primary palliative approach. For this reason, coupled with our key informant methodology, we suggest that our smaller sample size of 11 interviews is sufficient for the scope and aim of this paper (Guest *et al*., 2006; Malterud *et al*., 2016). Key informants were chosen in order to ensure a rich data set that stemmed from clinicians who were already tuned in to the forms of care relating to a primary palliative approach, and so our participants were not simply primary care clinicians in general but strategically selected interlocutors (Marshall, 1996). Moreover, while it is likely that further interviews would have revealed additional clinical minutia by probing the various idiosyncrasies of different practices, we argue that the higher-order themes and general trends developed through our analyses would not likely change or expand meaningfully.

To further ensure rigour, we conducted member checking with three participants who had expressed strong interest in follow-up during their interview. S.D. provided participants with a two-page summary of the coding framework and subsequent thematic analysis document and asked participants whether they felt their experiences were reflected by our analyses. Responses were brought back to the full research team to further refine codes and make minor alterations.

## Findings

Of the 11 interviewees, six were physicians, three were nurse practitioners, one was a registered nurse, and one was a registered practical nurse (Table 2). Eight participants were female. The ranges of clinical years at their current practices were 5–30 for physicians and 7.5–10 for nurses. All participants self-reported as practicing in urban/suburban settings, and all participants worked in different practices from one another. Eight participants practiced in a group setting and three in solo physician practices. All provided home visits.

**Table 2.** Interview participant characteristics (self-reported)

P*	Sex	Designation	Years at Clinic	Licensed	Population Type	Home Visits?	Practice Setting*
1	Female	MD	30	1985	Urban/suburban	Yes	Group physician practice
2	Female	MD	7	2005	Urban/suburban	Yes	Group physician practice
3	Female	NP	9	2001	Urban/suburban	Yes	University
4	Male	MD	10	2003	Urban/suburban	Yes	Group physician practice
5	Female	MD	5	2008	Urban/suburban	Yes	Group physician practice
6	Female	NP	8	2002	Urban/suburban	Yes	University
7	Male	NP	10	2005	Urban/suburban	Yes	University
8	Male	MD	15	1984	Urban/suburban	Yes	Solo physician practice
9	Female	RN	7.5	-	Urban/suburban	Yes	Solo physician practice
10	Female	MD	5	1994	Urban/suburban	Yes	Group physician practice
11	Female	RPN	10	1989	Urban/suburban	Yes	Solo physician practice

* All participants worked in different practices.

We have heuristically organized our interview findings and subsequent sub-themes under two main categories: (1) the identification of patients with PLLI and (2) the strategies used to assess and provide their care.

### Identification of patients with PLLI

Illnesses that our participants classified as common PLLIs included chronic obstructive pulmonary disease (COPD), heart failure and related complications, Parkinson’s dementia, amyotrophic lateral sclerosis, skin conditions, frailty and physical decline, pulmonary fibrosis, as well as psychosocial illnesses like loneliness and depression. Other common words used to signify patients with PLLI were ‘non-cancer’ and ‘non-curative’. Additionally, participants self-reported that they provided care for a range of 30–300 patients with PLLI depending on the size and age of the practice.

We identified four interrelated sub-themes with respect to the ways in which our key informant clinicians identified patients with PLLI: functional decline, change in needs, heightened acuity, and the specifics of a condition/diagnosis.

In identifying patients with PLLI, our participants emphasized the recognition of ‘baseline’ functional and psychosocial decline (e.g., increasing fatigue, depression, and anxiety, as well as diminishing social engagement), and an awareness that changes in patients’ daily routines and usual patient–practice interactions indicated a change in health care needs (e.g., a patient or family/caregiver was phoning the clinic more often, or conversely, they were suddenly ‘off the radar’, where participants ‘[had not] seen my patient in months’). Such changes in patient functioning and subsequent care needs were often identified by clear pattern changes to patient–practice interactions and the patient’s daily life more broadly:

So one of the first indications to me is when they’re not able to come to the office anymore. They can’t get out of bed, they’re too short of breath, caregivers can’t lift them, it’s too much for them to come out. (P2)

I have one patient with COPD, I mean a smoker who wouldn’t stop smoking, you saw him go downhill with his function, psychiatrically as well, depression, [affecting him and his] family. (P1)

Sudden acute changes in health status (a crisis) like exacerbating conditions, emergency visits, increased specialist care, patients suddenly requiring assistive devices, and the abrupt need for family and/or homecare support were reported as common triggers to develop a care plan and shift to a palliative oriented approach:

We are often triggered to these people by a panicked phone call or a hospital admission … a crisis or a set of crises …. They may have had this condition for years and it was stable and fine but when suddenly I see a change in their quality of life, that tends to be the first trigger. (P4)

Our participants also described an overall sense of increasing health complexity (e.g., comorbid conditions, diseases entering their later stages, heightened acuity) and the exhaustion of curative/preventative care options more generally:

We hit a point where we’re just sort of spinning our wheels, so to speak, and there is really no opportunity for improvement. And what happens is that health care providers start to feel helpless and frustrated and they start to have repeat conversations around what direction are we going to go, is there anything else we can do, what solutions can we explore? Finally, someone on the team will say ‘really, this is a life limiting illness and we need to adjust our expectations’. (P6)

The characteristics of a patient’s known diagnosis or chronic condition often led to a shift in care approach, for example, the onset of multiple comorbidities, when a disease is approaching its later stages and complications become severe, when life expectancy questions come to mind, and when medications no longer comfortably control a condition.

### Strategies to care for patients with PLLI

Three overarching themes and seven sub-themes were described by participants as strategies that assisted in providing a primary palliative approach to care. Many of these themes overlap and co-implicate one another, but we have separated them heuristically for the purposes of description.

First, our participants described an overarching theme of explicitly making a ‘commitment to care for patients and their families’. Specifically, they mentioned concretizing their relationship and pronouncing their responsibilities to the complex illness trajectories of PLLIs.

I tell them outright, whatever happens, good or bad, I’m going to be there for you …. I am going to do my best to help you. I try to get emotionally involved, because you’re caring for people, and you’ll make better decisions because you’ll know where they’re coming from. (P8)

The practices associated with concretizing their relationships helped to shape future communication channels and develop shared trust, facilitating the appropriate elicitation of care goals. Participants expressed that it was critical to encourage their patients to begin and lead a longitudinal conversation about their goals of care and to document these conversations consistently:

Having the conversation with the patient and clarifying early-on … what their desires are, who their power of attorney is [surrogate, substitute decision maker] … because a lot of patients don’t have that in place, or haven’t thought about it … communicating about these things [is] very helpful. (P2)

You have to talk to families about what to expect, and a onetime only conversation is never adequate to prepare people. They need ongoing support and ongoing preparation for what they are facing. (P6)

Finally, within this first theme, participants described shifting the place of care from office-based visits to home visits and being alert for this need of patients and families as illnesses progressed:

For some people I book regular home visits – if they’re at that point when patients are finding it difficult to come and see me, and often it is more near the end of life, but not necessarily. I had one lady who I was doing home visits for, for the last two years of life. Just because she couldn’t come and see me, so that was the best way to do it. (P5)

Second, our participants worked immediately to ‘broaden their care teams’ with any and all resources available in the context of each patient and family. The three sub-themes within this approach included bringing on interdisciplinary supports within the practice, leveraging informal caregivers into care routines right from the moment of identification, and engaging community supports. Specifically, participants expressed the necessity of broadening the care team and engaging in an individualized ‘team approach’ to care. This broadening of the care team included the leveraging of informal caregivers as well as identifying the family member who would be able to support the patient and maintain consistent communication with the practice:

[Identify] a committed family member. Things go well if you can ensure that patients have one family member that comes to every appointment. I find if I do that … I listen to them … [the care process] goes very well. (P1)

I’m a big believer in using other people as resources – you know, tapping into their expertise …. When you have more of the complicated issues, having an interdisciplinary team is amazing. (P9)

All participants reported that bringing on external community supports such as homecare organizations and pharmacists was critical to their care strategies and that educating their patients and their families about available services often increased their use. The majority of our participants suggested that formal care within the home should ramp up by engaging with homecare services and conducting frequent and temporally consistent home visits:

[During a home visit], I do an assessment of how’s everybody holding it together and managing with the crisis they’re dealing with. A quick assessment, you know, does the house look clean, does it look safe, and are we looking at proper food, how’s everybody coping. From there, it’s you know, blood pressure, physical assessment, what kind of physical stuff do we see going on. And we’ve been right up there as to hold your hand as you’re going [death]. (P11)

So when I go [to a home visit] it’s mainly conversation. I think the physical part, like the exam part, only takes up part of it, but it’s not the main component, a lot of it is talking to the patient, finding out where they are, what’s changed, how they’ve been, where things are at. A lot of talking to the caregiver, whoever they’re living with, and asking what’s going on. Medication review is a huge thing, getting out all the meds, what’s working, what isn’t, going through side effects, and dealing with that. Checking in on how the level of care is, is the caregiver still able to care, are we dealing with any burnout? (P2)

Third, once a committed relationship and mutual understanding about their patients’ condition was established and with a broadened care team in place, our participants then shifted to ‘proactive care practices’. Sub-themes included more proactive follow-up, more flexibility and iteration of the care agenda, and overall increased involvement such as telephone check-ins and spending more time with the patient during appointments:

One thing is, that the care from my perspective needs to shift into what I call ‘planned pro-active care’, you know, a shift into inviting patients and families to come in, and then we continue to book planned follow up. Not based on the patient going, ‘oh you know, my pain is worse and I’m not breathing as well’, but based on our understanding of how quickly they might progress or might change and then we see them weekly or monthly or every two months, but we start to plan to care. (P7)

Specific examples included booking appointments regularly and for longer durations, scheduling consistent home visits, and offering telephone support. These activities enabled the necessary increase in communication between patient/family/caregiver and clinic, between team clinicians, and with homecare.

Sometimes I start seeing people quite regularly and I ask them to check in with me because I notice that’s what works best for them. To do these regular check-in visits. They won’t bring up issues unless I have a pre-booked appointment. (P5)

Even if it’s a really slow progression, it might mean we’re only seeing them once a month, but if it speeds up we may actually go to weekly, so it’s really just meeting them where they need to be met. (P3)

We often intentionally book patients’ appointments for them, I mean, you know a lot of appointments are made when the patient perceives there’s a problem … but we often switch to intentionally booking appointments when we feel there’s discussions that need to take place around their care. (P6)

Being flexible and following the patient’s agenda helped to maintain a relational commitment to patients and their families:

Finding out the needs takes a few different visits, but really just inquiring step by step, and going at whatever level they’re at. Because even though we might anticipate some great needs down the road, try to meet them at the stage they are at. So if they’re still in transition, realizing there’s no curative, we’ll stay at that stage but try to be a little bit proactive in thinking about what we might need in the future. So it’s really just attending to them in every stage that they’re at. (P3)

Indeed, simply spending more time with patients after having identified them, including increased home visits, practice–patient communication, and a commitment to undertake more ‘upstream’ forms of care, acted as the foundation for our key informants’ successful care experiences.

## Discussion

This qualitative study of 11 primary care clinicians contributes to the practical clarity of a palliative approach in primary care. We have provided descriptions of the clinical strategies that our key informants employ to identify and care for patients with PLLI. Our findings revealed examples of clinical strategies that have worked to identify patients based on function and need, reaffirm the commitment to journey with the patient, broaden and facilitate communication with a care team, practice forms of ‘proactive’ care, and ensure early and continuous elicitation of care goals with patients and their families. The themes emerging from our findings align with the conceptual domains of a palliative approach, namely, an upstream orientation, adaptation of palliative approaches to varying illnesses and situations, and integration of a palliative approach into a generalist setting (Sawatzky *et al*., 2016).

Defining a palliative approach from the perspective of family medicine and primary care has been elusive because many still understand palliative care as separate from care in the general practice or family medicine setting, in the form of a specialty skill set. Reports that family physicians do not provide palliative care have prompted assessments of barriers to providing such care, citing that many feel ill-equipped due to lack of training, inadequate exposure to maintain skills, and overall perceived complex needs of patients at the end of life (Mitchell, 2002; Groot *et al*., 2005). A European study found that the most common reason reported by family physicians for their recently deceased patient not having received formal palliative care was the perception that the patient was already receiving adequate care and that they were more likely to report this reason for patients who were older and died in the community (Beernaert, Deliens, *et al*., 2015).

Our results about how primary care clinicians identified PLLI demonstrate the need to operationalize an upstream orientation as part of a palliative approach, specifically for primary care. The ways in which our participants identified PLLI were similar to published prognostic tools that function to trigger the initiation of palliative approaches (Gómez-Batiste *et al*., 2017; Murray *et al*., 2017) but the trigger for our participants was related to addressing patient needs based on long-standing knowledge of the patient rather than being alert to a prognosis, suggesting that further tailoring of approaches to specific practice settings may be needed.

Our study adds to previous research that has investigated family physicians’ and other primary care professionals’ roles in the care of patients with life-limiting illness. Beernaert *et al*. described clinicians who perceived their roles as medical expert, communicator, collaborator, and life-long learner (Beernaert, Van den Block, *et al*., 2015). In that study, health care professionals described how they approached care, but the emphasis was less on describing how they operationalized care processes and more on their perceived role. Our study contributes further to the knowledge of how upstream integration of a palliative approach can happen in primary care by describing in more depth the activities and guiding principles leveraged in the setting.

In a Canadian context, a commonly cited statistic that only one-third of patients receive palliative care is based on the understanding that such care is counted only if delivered by specialist teams (Shadd *et al*., 2013). Our results further support the suggestion of previous research that approaches that define the vision for family medicine such as continuity and comprehensiveness have much in common with a palliative approach (The College of Family Physicians of Canada, 2011; Beernaert, Van den Block, *et al*., 2015). While we did not ask for our participants’ views on the alignment of a palliative approach to the tenets of the family medicine discipline, our findings suggest that central to the provision of their care was that family medicine offered the longitudinal and relational engagement with patients that most contributed to timely and quality care throughout the often long-term progression of illness to end of life. Although we did not ask about provision of ‘palliative care’ in our interview questions, our participants realized that for some of their patients with PLLI, they were already providing palliative care, but they did not distinguish a separate approach based on the patient’s nearness to death. Understanding the current needs of patients with PLLI until the end of life as well as the capacity across our health care systems to meet these needs should be enhanced by a better understanding, articulation, and measurement of the contribution of the routine work of both primary care clinicians and palliative care specialists in providing a palliative approach.

### Limitations

There are some limitations in our study. It was conducted in one region, and given the diversity of clinical contexts across Canada and abroad, the results may not reflect general practice or family medicine in other jurisdictions. However, family medicine and primary care have in many countries similar roles in the health care system to Canada (Kringos *et al*., 2010). Moreover, because this study took place in predominantly urban/suburban settings in one area of Ontario, Canada, our findings may not be transferable to other settings, for example, rural regions.

## Conclusion

We found that our key informant primary care providers could articulate strategies for identifying and providing care to patients with PLLI that align to both the principles of family medicine and an upstream palliative approach to care, even though they did not describe a moment of sudden shift to palliative care. Indeed, at the core of our participants’ approaches to care for patients with PLLI were the practical clinical strategies and forms of care long since associated with the fundamentals of family medicine. Participants described how they identify patients with PLLI through changes in function and well-being that are recognized because of care continuity, and they responded by mobilizing and coordinating resources across practice, home, and community, as well as by engaging patients and family/caregivers in articulating their goals of care.

This study offers practical insights that can be used for the description, measurement, evaluation, and pedagogy of an evolving primary palliative approach. This description of the specific practices and clinical strategies relating to care of patients with PLLI may help clinicians, educators, and health system planners have an improved awareness of such practices as critical parts of a primary palliative approach to care. Future studies should direct their attention to validating these practices with respect to their potential impact on an integrated palliative approach across our health care system, as well as investigate the specific clinical facilitators and barriers to providing such care.
